# Supplementary material for: The role of cyclone activity in snow accumulation on Arctic sea ice
Source: Nat Commun. 2019 Nov 21;10:5285. doi: 10.1038/s41467-019-13299-8 (PMC6872656; doi:10.1038/s41467-019-13299-8)
Supplement: Supplementary file 2 — Supplementary Information [file 41467_2019_13299_MOESM2_ESM.docx]

Supporting information for “The role of cyclone activity on snow accumulation on Arctic sea ice”

M. A. Webster^1*^ et al.^2,3,4^

^1^University of Alaska Fairbanks, Geophysical Institute, 2156 Koyukuk Drive, Fairbanks, AK 99775, USA

^2^NASA Goddard Space Flight Center, 8800 Greenbelt Rd., Greenbelt, MD 20771, USA

^3^Earth System Science Interdisciplinary Center, University of Maryland, 5825 University Research Court Suite 4001, College Park, MD 20740, USA

^4^Jet Propulsion Laboratory, 4800 Oak Grove Dr., Pasadena, CA 91109, USA

M. A. Webster, University of Alaska Fairbanks, Geophysical Institute, 2156 Koyukuk Drive, Fairbanks, AK 99775, USA ([mwebster3@alaska.edu](mailto:mwebster3@alaska.edu))

Contents of this file

1. Supplementary Figures 1-5, Supplementary Tables 1-4, and Supplementary Discussions.

This auxiliary material contains five figures and four tables, followed by discussions of comparative analyses. These figures and tables are referred to in the main text.

**Supplementary Figures**


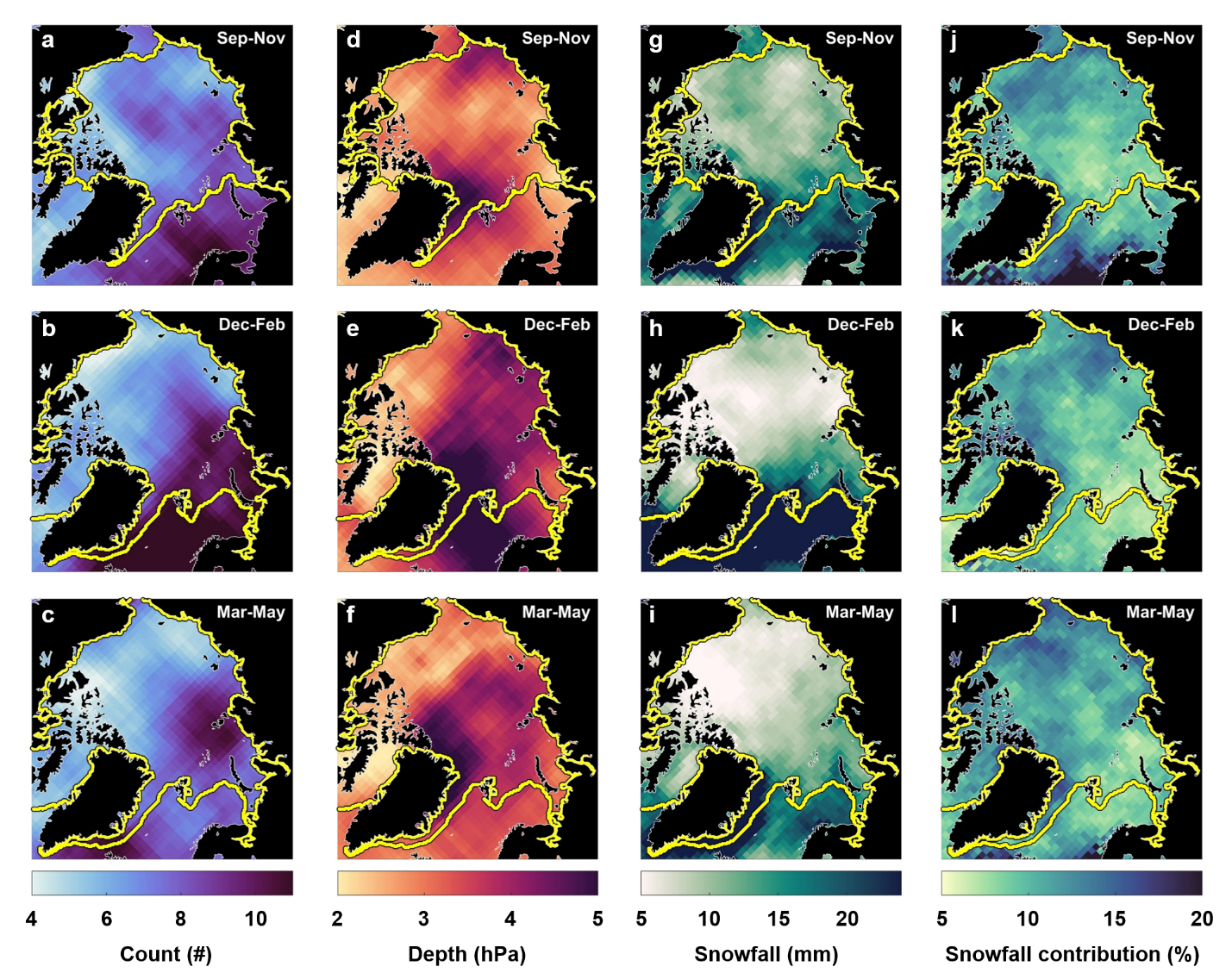


**Supplementary Figure 1 | The 1979-2016 inter-annual variability in cyclone variables.** The standard deviation in the 1979-20167 seasonal: a-c, cyclone count, which is the number of times a grid cell is encompassed by a cyclone’s area; d-f, cyclone depth, a metric for cyclone intensity that takes into account cyclone size and intensity; g-i, cyclone snowfall amount; and j-l, cyclone snowfall contribution, which is the percent contribution of snowfall from cyclones relative to the total snowfall. The 1979-2016 mean sea ice edge is outlined in yellow.


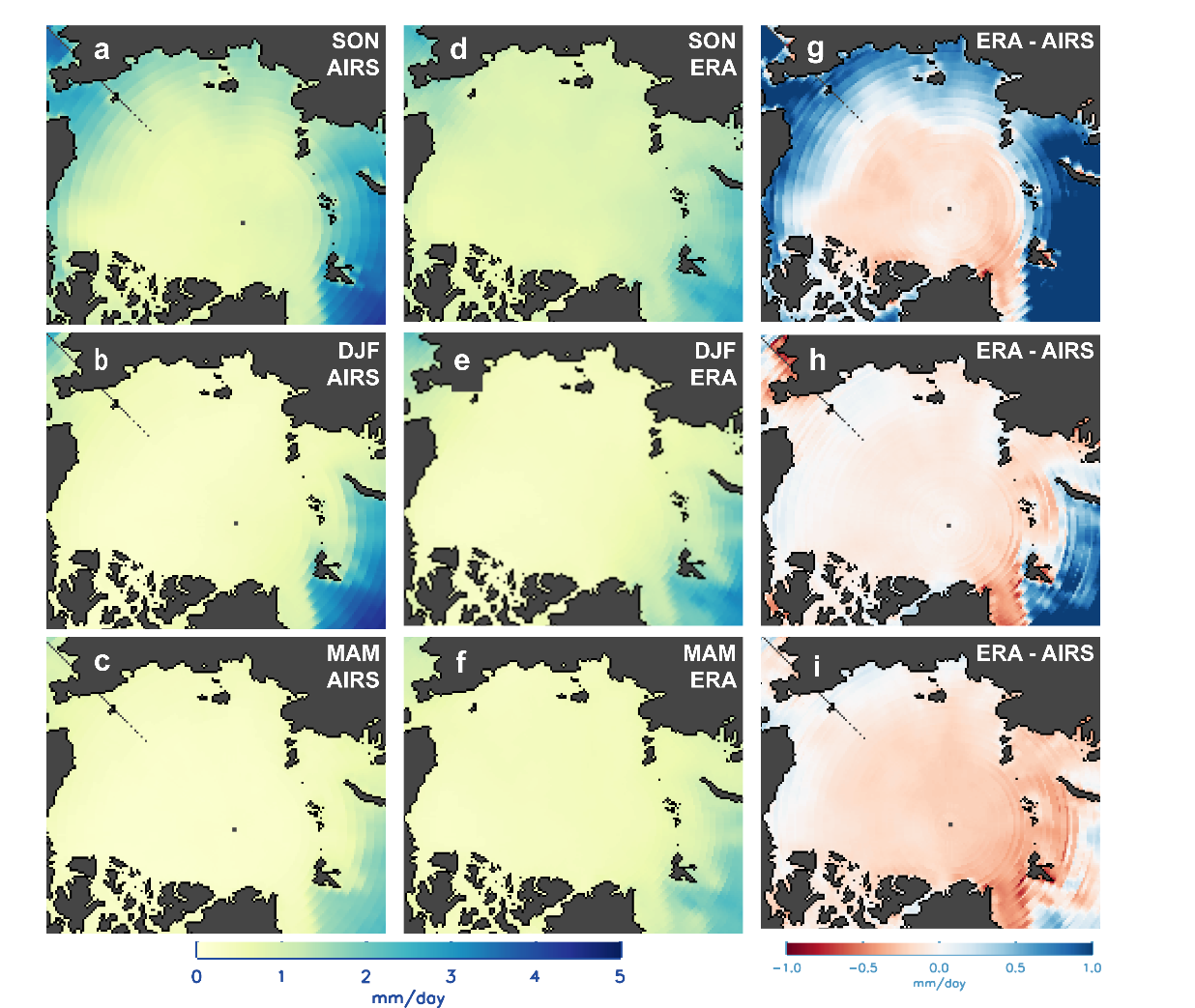


**Supplementary Figure 2| Comparison of AIRS and ERA-Interim snowfall.** A comparison of mean snowfall between (a-c) AIRS and (d-f) ERA-Interim for the autumn (September – November), winter (December – February), and spring (March – May) over the 2003-2016 period. The difference between ERA-Interim and AIRS is shown in panels (g-i).


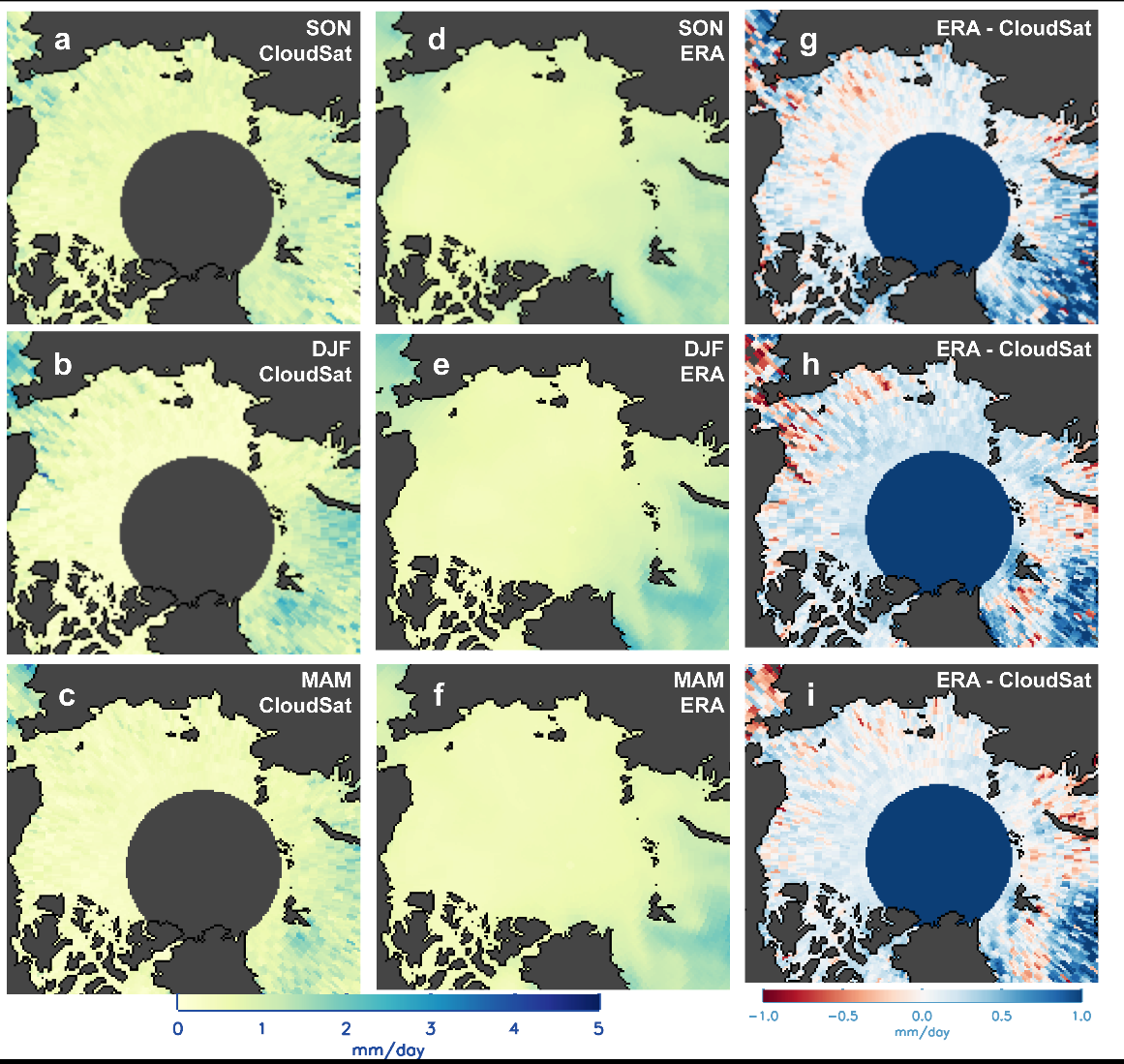


**Supplementary Figure 3 | Comparison of CloudSat and ERA-Interim snowfall.** A comparison of mean snowfall between (a-c) CloudSat and (d-f) ERA-Interim for the autumn (September – November), winter (December – February), and spring (March – May) over the 2003-2016 period. The difference between ERA-Interim and CloudSat is shown in panels (g-i).


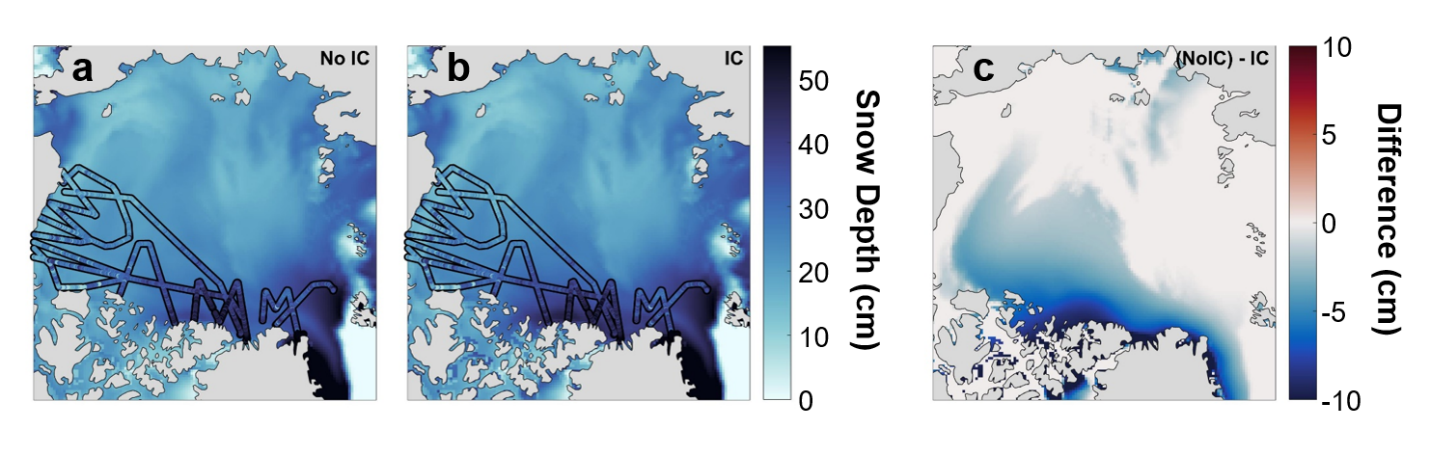


**Supplementary Figure 4 | Initial conditions of reconstructed snow depths.** The mean March-April snow depth distribution using (A) zero initial conditions, (B) the August climatology as initial conditions, and (C) the difference between the two reconstructions. Snow depths derived from the Operation IceBridge snow radar are overlaid in panels (A) and (B). All units are in centimeters.


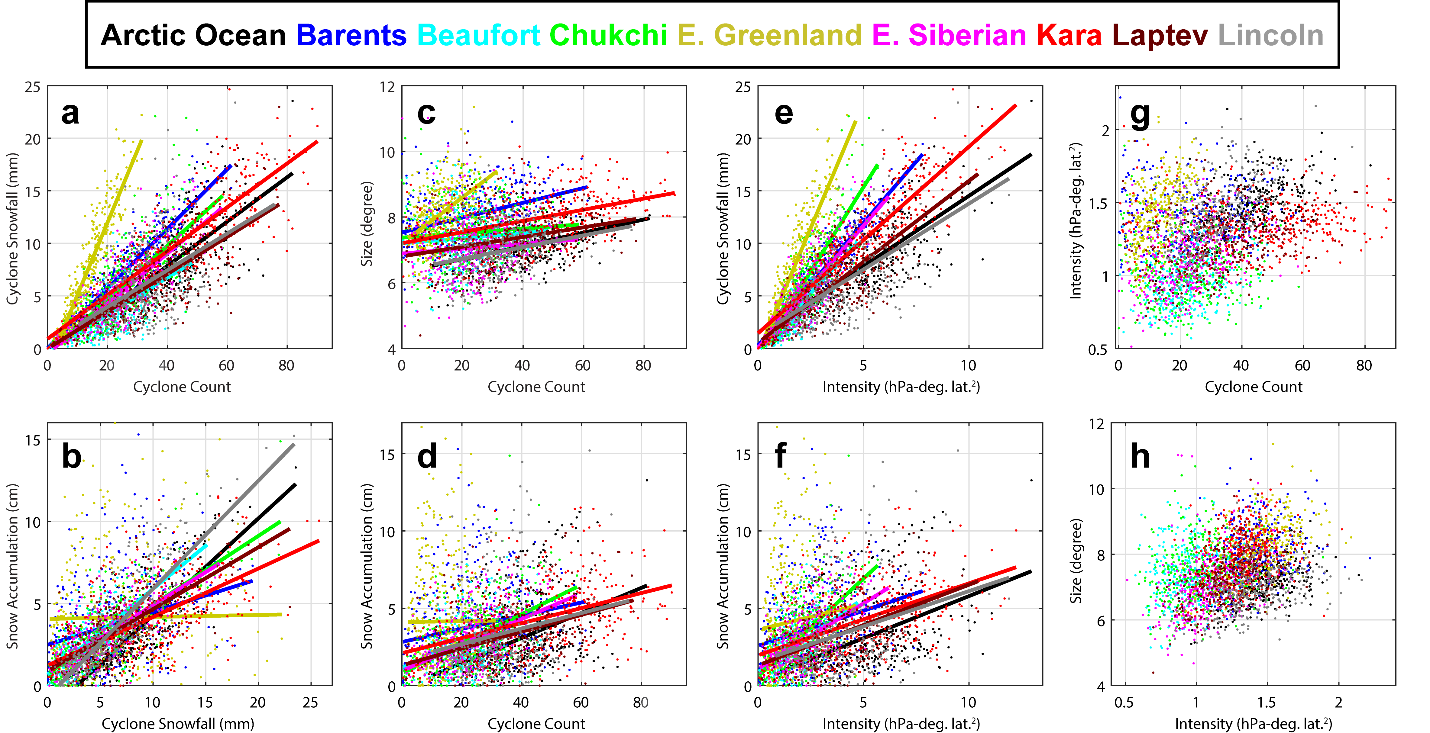
**Supplementary Figure 5 | The relationships between different cyclone variables and snow accumulation on a monthly basis.** The clustering of monthly data points is shown relative to the linear regressions for **(a)** cyclone snowfall and cyclone count, **(b)** snow accumulation and cyclone snowfall, **(c)** cyclone size (radius in degrees) and cyclone count, **(d)** snow accumulation and cyclone count, **(e)** cyclone snowfall and cyclone intensity, **(f)** snow accumulation and cyclone intensity, **(g)** intensity and cyclone count, and **(h)** cyclone size (radius in degrees) and cyclone intensity. Each color is representative of a region. Cyclone intensity vs. cyclone count and size had poor linear fits (panels g and h).

**Supplementary Tables**

| R^2^ of late sea-ice freeze-up | Snow accumulation | Total precipitation | Snowfall | Count | Size | Min. sea level pressure | Intensity |
| --- | --- | --- | --- | --- | --- | --- | --- |
| Lincoln | **-0.24*** | 0.06 | 0.04 | 0.06 | 0.01 | **0.12*** | 0.01 |
| E. Greenland | **-0.22*** | 0.00 | -0.08 | **-0.11*** | **-0.17*** | **0.13*** | -0.13 |
| Barents | **-0.49*** | -0.06 | **-0.30*** | -**0.20*** | -**0.18*** | **0.22*** | **-0.20*** |
| Arctic Ocean | **-0.49*** | **0.20*** | 0.07 | 0.03 | 0.01 | -0.02 | 0.02 |
| Kara | **-0.19*** | 0.05 | -0.01 | **-0.10*** | -0.09 | -0.09 | -0.08 |
| Laptev | -0.01 | 0.07 | 0.02 | 0.00 | 0.00 | 0.01 | 0.00 |
| E. Siberian | **-0.42*** | **0.10*** | 0.01 | 0.09 | 0.09 | -0.01 | 0.09 |
| Chukchi | **-0.20*** | 0.06 | 0.01 | 0.04 | 0.05 | -0.01 | 0.03 |
| Beaufort | **-0.37*** | 0.03 | -0.01 | 0.00 | 0.00 | 0.01 | -0.02 |
| *Arctic Average:* | *-0.29* | *0.06* | *-0.03* | *-0.02* | *-0.03* | *0.06* | *-0.03* |

**Supplementary Table 1 |** Linear regression results for late sea-ice freeze-up (independent variable) and the annual values of a number of (dependent) variables including: the annual snow accumulation, cyclone total precipitation, cyclone snowfall, cyclone density, cyclone size, the cyclone minimum sea level pressure, and cyclone intensity. The E. Greenland Sea does not have freeze-up dates. Thus, the regression in this region was not assessed. Results with at least 95% significance correlations are denoted with an asterisk (*).

| R^2^ of monthly snow accumulation | Univariate: cyclone count | Univariate: cyclone intensity | Univariate: cyclone snowfall | Univariate: cyclone size | Multivariate (a): count, intensity | Multivariate (b): count, intensity, size |
| --- | --- | --- | --- | --- | --- | --- |
| Lincoln | 0.03* | 0.07* | 0.71* | 0.03* | 0.07* | 0.12* |
| E. Greenland | 0.00 | 0.01 | 0.00 | 0.00 | 0.13* | 0.12* |
| Barents | 0.03* | 0.05* | 0.06* | 0.03* | 0.08* | 0.09* |
| Arctic Ocean | 0.15* | 0.20* | 0.72* | 0.11* | 0.17* | 0.28* |
| Kara | 0.15* | 0.21* | 0.33* | 0.15* | 0.22* | 0.22* |
| Laptev | 0.16* | 0.22* | 0.44* | 0.16* | 0.23* | 0.23* |
| E. Siberian | 0.27* | 0.33* | 0.58* | 0.28* | 0.31* | 0.31* |
| Chukchi | 0.24* | 0.35* | 0.55* | 0.23* | 0.35* | 0.35* |
| Beaufort | 0.11* | 0.23* | 0.58* | 0.08* | 0.27* | 0.28* |
| *Arctic Average:* | *0.13* | *0.19* | *0.44* | *0.12* | *0.20* | *0.22* |

**Supplementary Table 2 |** Linear regression results for the monthly snow accumulation (dependent variable) and the monthly cyclone measures of count, intensity, and snowfall (independent variables). Multivariate regression results for the monthly snow accumulation (dependent variable) predicted by models considering the independent variables a) monthly cyclone measures of count, intensity, and b) cyclone count, intensity, and size. The correlations listed are the results from the multivariate models. For the multivariate analysis, adjusted correlations are listed to account for more degrees of freedom. Results with at least 95% significance correlations are denoted with an asterisk (*).

| R^2^ of monthly cyclone snowfall | Cyclone count | Cyclone intensity |
| --- | --- | --- |
| Lincoln | 0.30* | 0.41* |
| E. Greenland | 0.85* | 0.87* |
| Barents | 0.91* | 0.93* |
| Arctic Ocean | 0.49* | 0.56* |
| Kara | 0.78* | 0.81* |
| Laptev | 0.58* | 0.61* |
| E. Siberian | 0.42* | 0.49* |
| Chukchi | 0.62* | 0.71* |
| Beaufort | 0.41* | 0.51* |
| *Arctic Average:* | *0.60* | *0.66* |

**Supplementary Table 3 |** Linear regression results for the monthly cyclone snowfall (dependent variable) and the monthly cyclone measures of cyclone count and intensity (independent variables). Results with at least 95% significance correlations are denoted with an asterisk (*).

| 2000A | 2007C | 2011A | 2012J | 2014C |
| --- | --- | --- | --- | --- |
| 2002A | 2007D | 2011I | 2012L | 2014E |
| 2003D | 2007E | 2011J | 2013B | 2014F |
| 2004C | 2008E | 2011K | 2013F | 2014I |
| 2004D | 2008F | 2012D | 2013G | 2015A |
| 2005F | 2009F | 2012G | 2013I | 2015D |
| 2006D | 2010A | 2012H | 2014B | 2015E |

**Supplementary Table 4** | Ice mass balance buoys from the Cold Regions and Research Engineering Laboratory that were used in the analysis^23,54^.

**Inter-annual variability in cyclone variables**Supplementary Figure 1 shows the spatial differences in the inter-annual variability in cyclone variables. Most notably, the spatial patterns do not coincide across all seasons. This suggests that several factors influence the inter-annual variability in cyclone characteristics.

**ERA-Interim snowfall and precipitation**

To explore possible biases in ERA-Interim snowfall, we compared the ERA-Interim precipitation and snowfall with NASA’s Atmospheric Infrared Sounder (AIRS) total precipitation and CloudSat’s snowfall, respectively.

Here, we compared the seasonal averages of total precipitation and their differences with AIRS data from 2003-2016 (Supplementary Figure 2). NASA’s Atmospheric Infrared Sounder (AIRS) precipitation data were also used to assess the magnitude of seasonal precipitation from ERA-Interim. AIRS is onboard the Aqua satellite and has a relatively long data record (September 2002 – present) with twice-daily global coverage, making it a temporally and spatially strong data set for comparison. The Level 3 precipitation product is a regression-based estimate, which computes precipitation from clouds and relative humidity information from the Level 2 infrared/microwave retrievals. AIRS is known to produce highly accurate temperature and humidity profiles, even under cloudy conditions, which is important in the Arctic where clouds are prevalent^37^. Temperature and humidity from AIRS have also been assessed along with ERA-Interim in the Arctic and show good agreement with *in situ* observations and are therefore deemed reliable for this analysis^36^.

In all seasons over the sea ice pack, the total precipitation between AIRS and ERA-Interim are very similar, with ERA producing slightly more precipitation over the sea ice pack, specifically in the north Atlantic region surrounding Svalbard in winter and spring (Supplementary Figure 2). Over the sea ice pack, ERA-Interim is known to have a positive humidity bias when compared to *in situ* data^48^, which could result in larger estimates of precipitation over the sea ice pack relative to AIRS data. In the ice-free regions of the north Atlantic, AIRS tends to produce more precipitation than ERA-Interim. Overall, the regions near the sea ice edge or in the marginal ice zone have the largest discrepancies. These differences between products could be due to the poor reproduction of evaporation by ERA-Interim^36^, errors in the emissivity returns from AIRS in those areas where cloud tops and sea ice produce similar returns, erroneously affecting the retrievals, or both. Although there is strong agreement between products, their differences provide further guidance on the potential biases in ERA-Interim precipitation.

**Initial conditions in snow depth reconstruction**

A factor to consider in the snow depth reconstruction is the duration of the snow cover during the summer melt season. For the purpose of this analysis, the initial conditions of the snow depth reconstruction started with a snow-free sea ice of each September to investigate the effects of cyclone activity on snow accumulation. However, data from the drifting ice stations in 1954-1991 indicate that snow sometimes persisted throughout the summer melt season, and, relative to the contemporary period, had a longer seasonal duration^11^. Indeed, snow was observed throughout the summer season during the SHEBA campaign, albeit being limited to drifts behind ridges^13^. What we can qualitatively deduce from the limited observations and remote sensing products available is that the amount of snow that survives the summer melt season has decreased in recent decades. High-resolution satellite imagery from the Global Fiducials Library [usgs.gfl.gov], previous works of melt pond distributions^61^ and AVHRR surface albedo^62^ collectively point to a summer sea ice cover that has substantial melt pond coverage and a lower albedo than the 1954-1991 climatology^11^, which suggests that less snow-covered sea ice is present. The lack of observations of snow in summer and the unknown rate of change in the duration of summer snow over the last several decades poses a challenge in modeling snow depth during the melt season^6^. As a general example, the results of applying the August climatology^12^ as initial conditions in the snow depth reconstruction is shown in Supplementary Figure 4.

**Statistical analyses**

Cyclone variables such as count and snowfall can co-vary (Supplementary Figure 5a,e). However, the correlations are regionally dependent and it is important to understand the influence of each cyclone variable on snow accumulation in a univariate framework. Our analysis of the data characteristics suggests that cyclone count, intensity, and size largely do not exhibit collinearity (Supplementary Figure 5c,g,h). Therefore, in the climatology, more cyclone events do not equate to stronger or larger cyclones and these variables can be considered in a multivariate framework. Prevailing atmospheric conditions and oscillations may influence interannual variability in cyclone characteristics. However, the univariate and multivariate regression analysis does not consider the correlation in cyclone variables over time, but explores the relationships between individual monthly measures of cyclone characteristics and the snow accumulation. While some resulting correlations are small, they are still statistically significant given the large sample size (336 or 456). Nevertheless, we note that some individual cyclone characteristics do not have strong relationships with snow accumulation given the small correlations. For the trend analysis, we explored linear, cubic, and exponential regressions for all variables, across all regions. Linear regressions exhibited the strongest fits with the data (i.e. lowest root-mean-squared errors) with only two exceptions. The exceptions were total regional snowfall and total precipitation, which cubic and exponential models yielded the lowest RMS errors, respectively. Given that the scope of the analysis does not focus on trends in total snowfall or total precipitation, we deferred to using linear regressions for all variables in the trend analysis for consistency in approach.
